# Iatrogenic tension pneumothorax developed during ventriculo-peritoneal shunt surgery and detected shortly before extubation

**DOI:** 10.1186/s40981-018-0177-y

**Published:** 2018-05-12

**Authors:** Yoshiyasu Hattammaru, Tomasz Hascilowicz, Isao Utsumi, Yuichi Murakami, Oumi Sachiko

**Affiliations:** 1 Department of Anesthesiology, The Jikei University School of Medicine Hospital, 3-19-18, Nishi-shimbashi, Minato-ku, Tokyo, 105-8471 Japan; 2 Department of Anesthesiology, Jikei University Daisan Hospital, 4-11-1, Izumihon-cho, Komae-shi, Tokyo, 201-8601 Japan

## To the editor

Few reports on iatrogenic pneumothorax developed during/after a ventriculo-peritoneal (VP) shunt surgery exist in the literature, but none discusses time of pneumothorax detection. In the presented case, a post-operative review of electronic anesthesia records indicated that diagnosis could have been made earlier and that tension pneumothorax could have been avoided.

### Case presentation

A 48-year-old man (158 cm, 65 kg, BMI 26.04) who had undergone clipping after subarachnoid hemorrhage was hospitalized for convulsions at 5 months following initial surgery. VP shunt operation was scheduled. Chest CT showed no pulmonary bullae or blebs before surgery.

Induction of general anesthesia was uneventful. At 140 min, the anesthesiology resident noticed an increase in airway pressure (AP) (to 25 cmH_2_O) and the attending decided to proceed with anesthesia since no abnormalities on the patient side (change in pulse oximetry readings or abnormal sounds on auscultation) or on the circuit side (endotracheal tube occlusion, kinking, anesthetic machine malfunction) were found. Arterial blood gas analysis showed pH 7.444, PaCO_2_ 35.2 mmHg, PaO_2_ 147 mmHg, and SaO_2_ 98.4%. Peak pressure stayed at 25–29 cmH_2_O. The attending concluded that increased AP was caused by patient’s position and/or patient’s body weight.

**Fig. 1:**
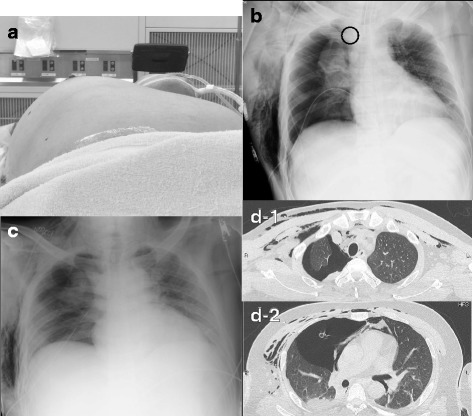
Postoperative images. **a** Significant chest distention on the right side. The photograph was taken after needle thoracentesis. **b** Chest X-ray showing right pneumothorax, heart shifted to the left, massive subcutaneous emphysema, and malpositioned chest drainage tube, which had to be re-positioned. **c** Correct placement of the drainage tube. **d-1**, **d-2** Chest CT showing subcutaneous and mediastinal emphysema, right pneumothorax, and no bullae or blebs

The surgery ended 40 min later and the asymmetrically deformed patient’s chest with distention on the right side was noticed after removal of surgical drapes. Soon after returning the patient’s head into the straight position, bucking occurred. The AP immediately increased and oxygen saturation dropped to 70%. The attending suspected tension pneumothorax, confirmed the diagnosis by absence of lung sliding on ultrasonography, and performed needle thoracentesis with an 18-G intravenous catheter under ultrasound guidance (Fig. 1a).

Following decompression, oxygen saturation returned to 100%. A chest X-ray taken a few minutes later revealed massive subcutaneous emphysema, right pneumothorax, and mediastinal shift to the left (Fig. 1b, c). After insertion of a chest drainage tube, the patient was extubated and transferred to the intensive care unit. Continuous air leakage was observed for few days postoperatively indicating intra-operative iatrogenic pleural and lung injury. The chest tube was removed on POD7. The patient was discharged and transferred to a rehabilitation hospital for further treatment on POD15.

### Discussion

In the literature, in most reported cases, pneumothorax occurred after ventriculo- or subduro-peritoneal shunt surgery [1–5], but in our case, tension pneumothorax occurred shortly before extubation. Time of pneumothorax detection is crucial for early diagnosis and treatment, and early detection could be facilitated by careful examination of electronic anesthesia records. In our case, the assessment indicated that the diagnosis could have been made earlier and tension pneumothorax progression could have been avoided—the resident reported AP changes 30 min after AP had actually begun to increase (Additional file 1), and timing of AP increase coexisted with surgical maneuvers in the neck region. The pleural tear caused by the passer might have acted as a check valve before returning the patient’s head position. Ultrasonography was useful to confirm the diagnosis and guide thoracentesis [6].

## Additional file

Additional file 1: Electrical Chart. (PDF 73 kb)
