# Supplementary material for: Iatrogenic tension pneumothorax developed during ventriculo-peritoneal shunt surgery and detected shortly before extubation
Source: JA Clin Rep. 2018 May 12;4:39. doi: 10.1186/s40981-018-0177-y (PMC6967180; doi:10.1186/s40981-018-0177-y)
Supplement: Supplementary file 1 — Electrical Chart. (PDF 73 kb) [file 40981_2018_177_MOESM1_ESM.pdf]

Appendix 1.

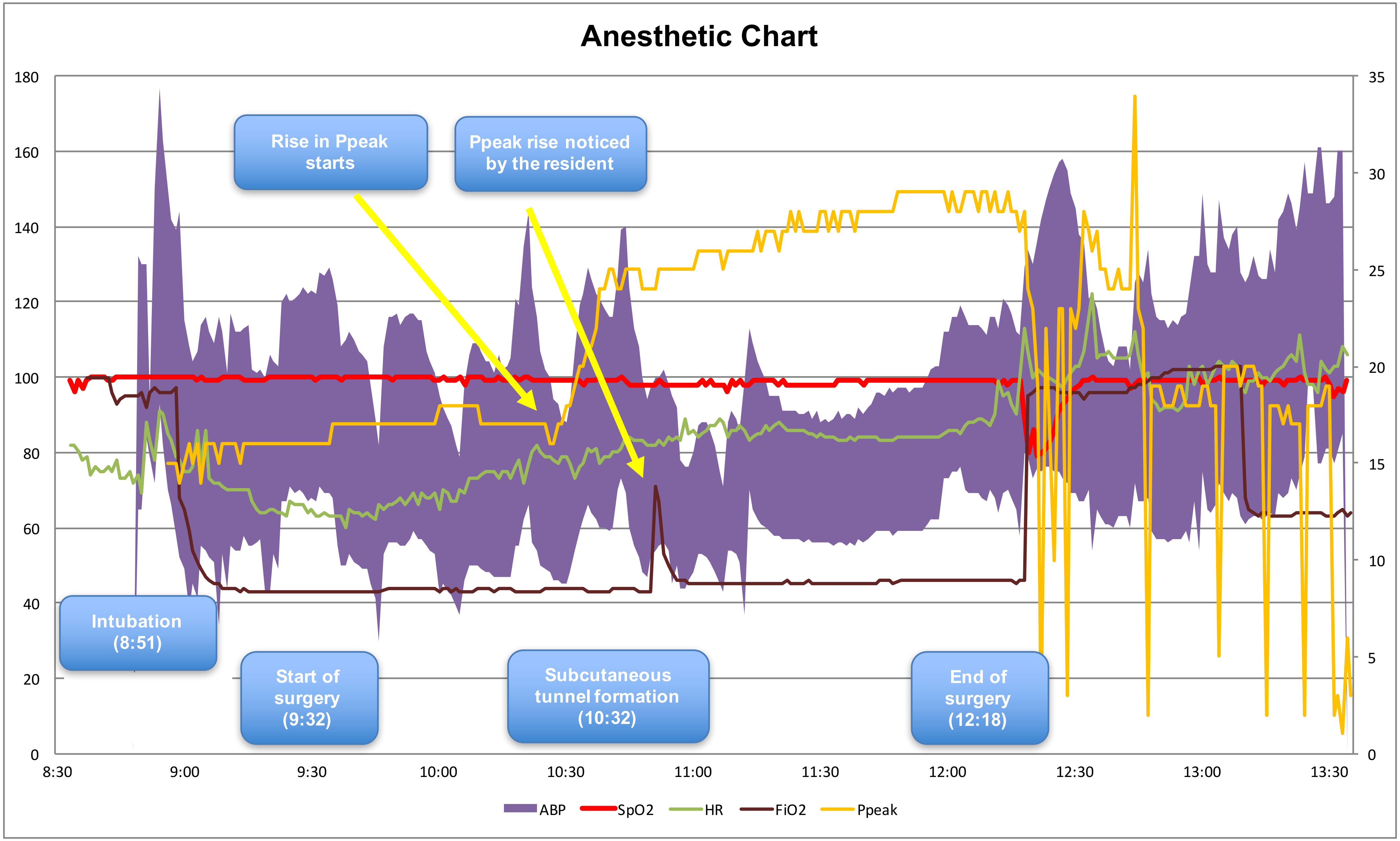

The airway pressure begins to rise up shortly after the start of subcutaneous tunnel making procedure. The resident notices the raised Ppeak pressure 30 minutes later prompted by the drop in oxygen saturation values but does not examine the trend data. To correct desaturation, he increases the concentration of inhaled oxygen and operation is being continued. However, another drop in saturation values is observed after the end of operation and removal of the surgical drapes. This corresponds with the highest Ppeak pressure values and development of tension pneumothorax. The gradual rise in Ppeak pressure had not been assessed by either anesthesia resident or attending anesthesiologist. A timely survey of the trend data could have prevented development of the tension pneumothorax.
